# Health outcomes across socioeconomic strata B, C, and DE among Brazilian adults living in moderate social vulnerability

**DOI:** 10.3389/fpsyt.2026.1849690

**Published:** 2026-07-03

**Authors:** Camilla Ytala Pinheiro Fernandes, Cristiane Maria Gonçalves, Lucas Melo Neves, Thais Reimberg, Patricia Colombo-Souza, Jane de Eston Armond, Natália Pinheiro Fabricio Formiga, Saulo Gil

**Affiliations:** 1 Santo Amaro University, Post-graduate Program in Health Sciences, Sao Paulo, Brazil; 2 Programa de Atividades Esportivas Extensivas à Comunidade – PAEC - Santo Amaro University, Rua Professor Enéas de Siqueira Neto, São Paulo, Brazil; 3 Bipolar Disorder Program (PROMAN), Department of Psychiatry, University of São Paulo Medical School, São Paulo, Brazil; 4 Physical Activity, Sport and Mental Health Laboratory (LAFESAM), São Paulo State University (UNESP), Institute of Biosciences, Department of Physical Education, Rio Claro, São Paulo, Brazil; 5 State University of Ceara, Fortaleza, Ceara, Brazil; 6 Applied Physiology and Nutrition Research Group - Center of Lifestyle Medicine, Faculdade de Medicina FMUSP, Universidade de São Paulo, Sao Paulo, Brazil

**Keywords:** cardiometabolic risk, inequality, mental health, poverty, quality of life

## Abstract

**Objectives:**

This study examined whether socioeconomic status was associated with anxiety symptoms, depressive symptoms, BMI, waist-to-hip ratio, and quality of life among Brazilian adults living in areas of moderate social vulnerability. In addition, we described anxiety and depressive symptoms, BMI and waist-to-hip ratio, and quality of life in individuals living in moderate social vulnerability.

**Methods:**

This is a cross-sectional study. In a socially vulnerable cohort, interviews captured demographics, comorbidities, medications, anxiety and depressive symptoms, and quality of life, followed by measurement of anthropometric characteristics.

**Results:**

Among 299 socially vulnerable adults, 8% had moderate–severe depressive symptoms and 7% had moderate–severe anxiety symptoms; ~50% showed increased risk of cardiometabolic diseases (i.e., waist-to-hip ratio greater or equal to 0.90 for men and 0.85 for women, respectively). Poor quality of life affected 4–12% across domains. Mental health, anthropometrics (waist-to-hip ratio, BMI), increased risk of cardiometabolic diseases and quality of life in physical, social and environmental domains did not differ by socioeconomic status (B, C, DE; all P>0.05). Poor psychological quality of life was more frequent among participants in higher socioeconomic status (B: 8%; C: 6%; DE: 4%, P = 0.0157). Linear regression analyses showed no statistically significant differences across socioeconomic status in depressive symptoms, anxiety symptoms, BMI, waist-to-hip ratio, or quality of life scores in any domain (all P>0.05).

**Conclusions:**

Our findings suggest that, among Brazilian adults living in moderate social vulnerability and classified within socioeconomic status B, C, and DE, mental health, BMI, waist-to-hip ratio, and quality-of-life indicators were similar across socioeconomic strata. However, these results should be interpreted as reflecting intra-group socioeconomic differences within a moderately vulnerable population and should not be generalized to individuals from the highest socioeconomic status.

## Introduction

Mental disorders, particularly anxiety and depression, are highly prevalent in the population, affecting a wide range of ages, both sexes, and distinct regions around the world (1). According to the 2019 Global Burden of Diseases, Injuries, and Risk Factors Study, anxiety and depressive disorders rank among the most disabling mental conditions and are listed as leading causes of disease burden worldwide (2). Furthermore, evidence has shown a marked increase in mental disorders following the COVID-19 pandemic (3–5). In Brazil, for instance, the prevalence of anxiety and depressive disorders rose by more than 20% between 2019 and 2020 (3).

In general, anxiety and depressive disorders are associated with an increased risk of mortality (6–8), but it is noteworthy that subclinical forms of these conditions (i.e., individuals who present symptoms but do not meet the full diagnostic criteria may also be at significant health risks (9, 10). Excess mortality in individuals with mental disorders extends far beyond the well-recognized risks of suicide and accidental injuries. It is also driven by a disproportionate burden of communicable and, most critically, non-communicable diseases (7, 8, 11). Among these cardiometabolic diseases emerge as the leading cause of premature death, affecting not only those with diagnosed mental disorders (7) but also individuals experiencing subclinical forms (10). This underscores the need for early detection of risk factors for both mental disorders and cardiometabolic diseases, enabling timely interventions and reducing the burden of morbidity and mortality.

Social vulnerability is a multidimensional concept that encompasses economic, social, environmental, and psychosocial challenges such as low socioeconomic status, limited educational opportunities, unstable housing, restricted access to healthcare, and reduced social support that collectively heighten exposure to stress and accelerate the onset of disease (12). Although findings are not entirely consensual, previous studies have shown that social vulnerability is associated with an increased risk of mental disorders (e.g., anxiety and depression) (13–15), cardiometabolic risk factors (e.g., obesity and waist circumference) (16, 17) and diabetes‐related cardiovascular mortality (18). Moreover, individuals living in social vulnerability often report poor quality of life (19–21). Although it is reasonable to assume that individuals living in moderate social vulnerability are similarly exposed to risk factors for mental and cardiometabolic diseases, additional factors, such as socioeconomic status (assessed by accumulation of consumer goods, household characteristics, access to public services, and the educational level of the head of household), may further exacerbate these conditions; however, little is known about how these combined factors manifest in this population.

This study examined whether socioeconomic status was associated with anxiety symptoms, depressive symptoms, BMI, waist-to-hip ratio, and quality of life among Brazilian adults living in areas of moderate social vulnerability. In addition, we described anxiety and depressive symptoms, BMI, waist-to-hip ratio, and quality of life in Brazilian adults living in moderate social vulnerability.

## Methods

### Study design and participants

This is a cross-sectional study conducted at Santo Amaro University (Sao Paulo, Brazil) between March 2024 and March 2025. Participants were recruited through university community programs and social media and were subsequently invited to attend the university for in-person assessments. Inclusion criteria were: (i) individuals aged over 17 years and; (ii) living in areas classified as moderate social vulnerability, defined by a score between 0.301 and 0.400 on the Social Vulnerability Index (*Índice de Vulnerabilidade Social* [IVS] developed by the Institute for Applied Economic Research in Brazil (22). Exclusion criteria were: (i) cognitive impairment that precluded the participant from understanding the study procedures or providing written informed consent; (ii) physical, cognitive, or communication limitations that prevented completion of the study assessments, even with reasonable support; and (iii) cancer diagnosis within the past 5 years.

Participants underwent a semi-structured interview to record sociodemographic characteristics (i.e., age, sex, marital status, ethnicity, family income), comorbidities, use of medications, anxiety and depressive symptoms and quality of life status. Subsequently, anthropometric characteristics (i.e., body weight, height, waist and hip circumference) were assessed.

This study was approved by the local Ethics Committee. All participants provided written informed consent before entering the study. This manuscript was reported according to the Strengthening the Reporting of Observational Studies in Epidemiology (STROBE) (23).

### Socioeconomic status

Socioeconomic status was estimated using the Brazilian Economic Classification Criteria developed by the Brazilian Association of Research Companies (*Associação Brasileira de Empresas de Pesquisa* - ABEP). This classification system evaluates socioeconomic status based on the accumulation of consumer goods, household characteristics, access to public services, and the educational level of the head of household. The total number of points is used to place the individual in a socioeconomic status category designated as A, B (i.e., B1, B2), C (i.e., C1, C2), DE, where A is the highest and DE is the lowest category (24).

### Depressive and anxiety symptoms assessment

The anxiety and depressive symptoms were assessed using the Beck Depression Inventory (BDI) (25) and Beck Anxiety Inventory (BAI) (26). Both questionnaires are composed of 21 multiple-choice statements, each with four possible answers (0–3). Thus, the final score ranges from 0 to 63 points. Both BDI and BAI were validated for the Brazilian population (27, 28). Participants were classified as having minimal (scores < 14), mild (scores 14-19), moderate (scores 20-28), and severe (scores > 28) depressive symptoms. Anxiety symptoms were classified as having minimal (< 8), mild (8–15), moderate (16–25),, and severe (> 25), respectively.

### Quality of life assessment

Quality of life was assessed using the World Health Organization Quality of Life (WHOQOL-bref) (29). This questionnaire consists of 26 questions, the first of which refers to quality of life in general, the second to satisfaction with one’s own health and the rest are divided into the physical, psychological, social and environmental domains. Scores range from 0 to 100 points, with higher scores indicating better quality of life. Individuals who scored below 50 points in each domain were classified as having a poor quality of life in that domain.

### Body mass index and waist-to-hip ratio assessment

Body weight was assessed on a calibrated digital scale and height was evaluated with the aid of a stadiometer, from which BMI was calculated (i.e., BMI = kg/m^2^). Waist and hip circumference were assessed using a measuring tape. Waist circumference was measured at the narrowest point between the lower ribs and the hips, while hip circumference was measured at the widest point of the hips when viewed from the side, from which waist-to-hip ratio was determined. Individuals with a waist-to-hip ratio greater than or equal to 0.90 for men and 0.85 for women were classified as having an increased risk of cardiometabolic diseases (30).

### Statistical analysis

The sample size was determined *a priori*, with the aid of the G-Power software (Version 3.1.9.2, University of Kiel, Germany), assuming a power (1 – β error) of 0.95 and α error of 0.05. The calculation was based on Analysis of variance (ANOVA) (Fixed effects, omnibus, one-way). We utilized an arbitrary and effect size of 0.25 [medium effect size according to Cohen (31)] for sample-size estimation. The total sample size returned by the software was 252 participants.

Data are presented as absolute (n) and relative (%) frequency, means ± SD. Data normality was determined via Shapiro-Wilk test and visually checked with histograms. Then, a one-way analysis of variance (ANOVA) was employed to compare anxiety and depressive symptoms, body mass index, waist-to-hip ratio and quality of life scores in each domain according to socioeconomic status. In case of significant F-values, *post-hoc* tests with Tukey’s adjustment were performed for multiple comparisons. Possible between-group differences (socioeconomic status [i.e., A, B, C and DE]) in the frequency of individuals having moderate/severe anxiety and depressive symptoms, obesity (BMI ≥ 30) and poor quality of life in each domain (i.e., score < 50) were assessed using contingency tables and Chi-square tests.

Adjusted linear regression models were utilized to verify possible associations between socioeconomic status and health outcomes. Adjusted linear regression model was adjusted by age (as continuous), ethnicity [white, black and pardo], sex [male or female], marital status [single, married, divorced and widowed], hypertension [yes or no], and type II diabetes [yes or no]. Beta coefficients were calculated along their corresponding 95% confidence intervals (95%CI). To account for multiple comparisons across regression models, P values for socioeconomic status terms were adjusted using the false discovery rate method according to Benjamini and Hochberg. Significance level was set at P ≤ 0.05. All analyses were performed in the statistical environment R (version 3.5.3; R Core Team 2020).

## Results

Two hundred and ninety-nine individuals were evaluated. Overall, the sample consists of individuals of both sexes (81% female) aged 57 ± 16 years. Fifty percent of the participants were married, 26% were single, 11% were divorced, and 13% were widowed. Frequency of ethnicity of individuals white, black or pardo (term used in Brazilian Portuguese, meaning “mixed ethnicity, “ according to the Brazilian Institute of Geography and Statistics) were 66%, 32%, and 2%, respectively. Regarding family income, 52% of individuals reported earning less than US$267.85, 14% reported earning between US$267.85 and US$535.70, 25% reported earning between US$535.70 and US$863.55, and 5% reported earning more than US$863.55. Additionally, 4% of individuals chose not to disclose their family income. Prevalence of systemic arterial hypertension, or obesity, or type 2 diabetes were 44%, 44%, and 22%, respectively. **Table 1** details demographic and clinical characteristics of the individuals.

**Table 1:** Demographic, socioeconomic and clinical characteristics of individuals living in areas of moderate social vulnerability.

Outcomes	n = 299
Age, years	57 ± 16
Sex, n (%)	
Female	243 (81%)
Male	56 (19%)
Marital status, n (%)	
Single	79 (26%)
Married	148 (50%)
Divorced	34 (11%)
Widowed	38 (13%)
Ethnicity, n (%)	
White	197 (66%)
Black	95 (32%)
Pardo^a^	7 (2%)
Family Income, n (%)	
< US$ 267.85	154 (52%)
US$ 267.85 to US$ 535.70	43 (14%)
US$ 535.70 to US$ 863.55	74 (25%)
> US$ 863.55	15 (5%)
Refrained from reporting	13 (4%)
Comorbidities, n (%)	
Systemic arterial hypertension	131 (44%)
Type 2 diabetes	66 (22%)
Obesity (BMI ≥30 kg/m²)	132 (44%)
Medication, n (%)	
β-blockers	13 (4%)
Metformin	20 (7%)
ACE inhibitor	13 (4%)
Angiotensin II receptor antagonism	43 (14%)
Diuretics	30 (10%)

a = Pardo is the exact term used in Brazilian Portuguese, meaning “mixed ethnicity, “ according to the Brazilian Institute of Geography and Statistics; ACE, angiotensin-converting enzyme.

Frequency of the individuals reporting severe, moderate, mild and minimal depressive symptoms were 2%, 6%, 11%, and 81%, respectively. For anxiety symptoms, 1% of participants reported severe symptoms, 6% reported moderate symptoms, 35% reported mild symptoms, and 58% reported minimal symptoms. Fifty percent of the individuals exhibited an increased risk of cardiometabolic diseases (i.e., waist-to-hip ratio greater or equal than 0.90 for men and 0.85 for women). The prevalence of the individuals reporting poor quality of life in the physical, psychological, social and environment domains were 6%, 4%, 9%, and 12%, respectively.

**Figure 1:**
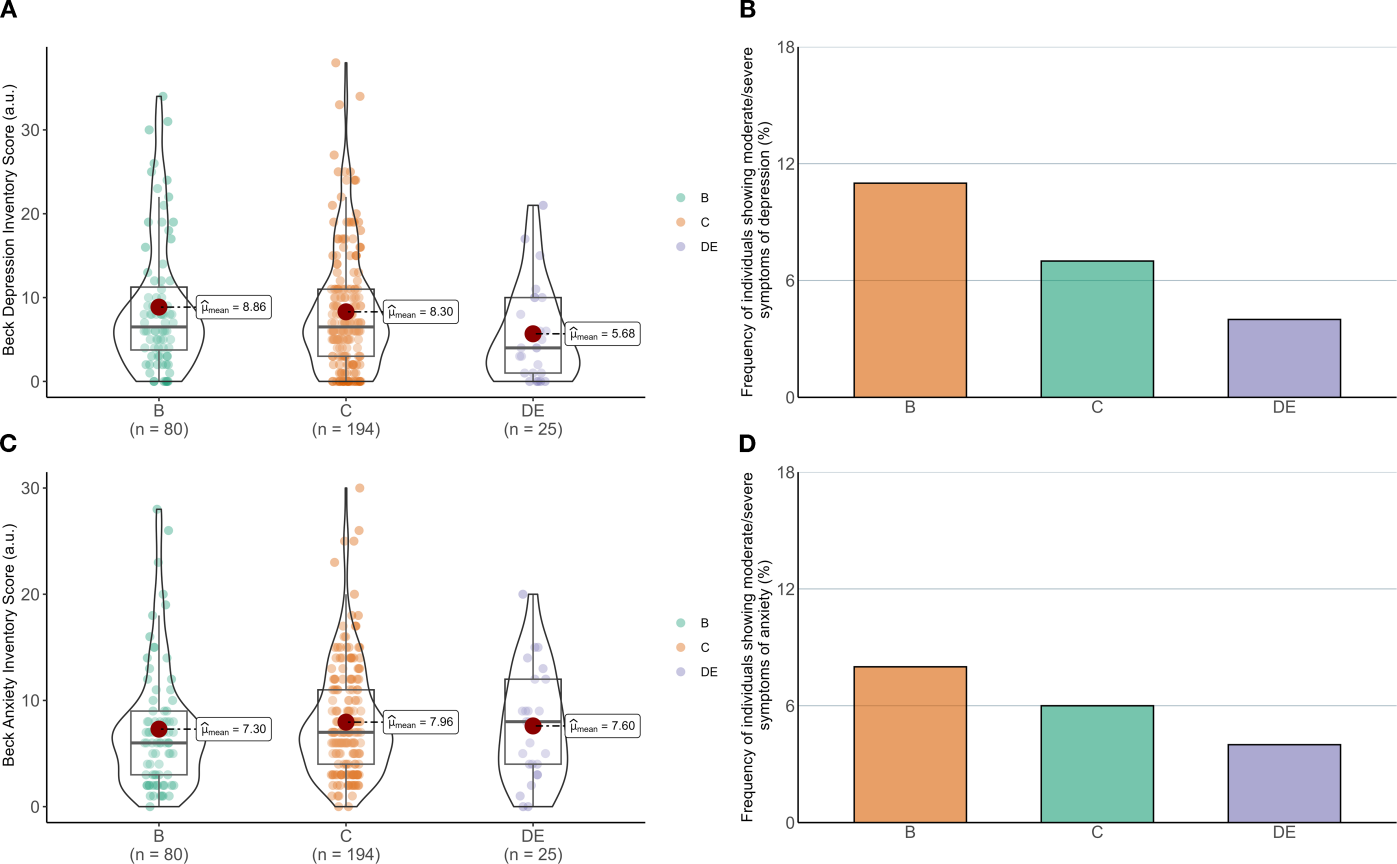
Symptoms of depression and anxiety according to socioeconomic status. Panel **(A)** indicates symptoms of depression according to socioeconomic status; Panel **(B)** indicates the frequency of individuals having moderate/severe symptoms of depression according to socioeconomic status; Panel **(C)** indicates symptoms of anxiety according to socioeconomic status; Panel **(D)** indicates the frequency of individuals having moderate/severe symptoms of anxiety according to socioeconomic status. a.u.: arbitrary units.

**Figure 2:**
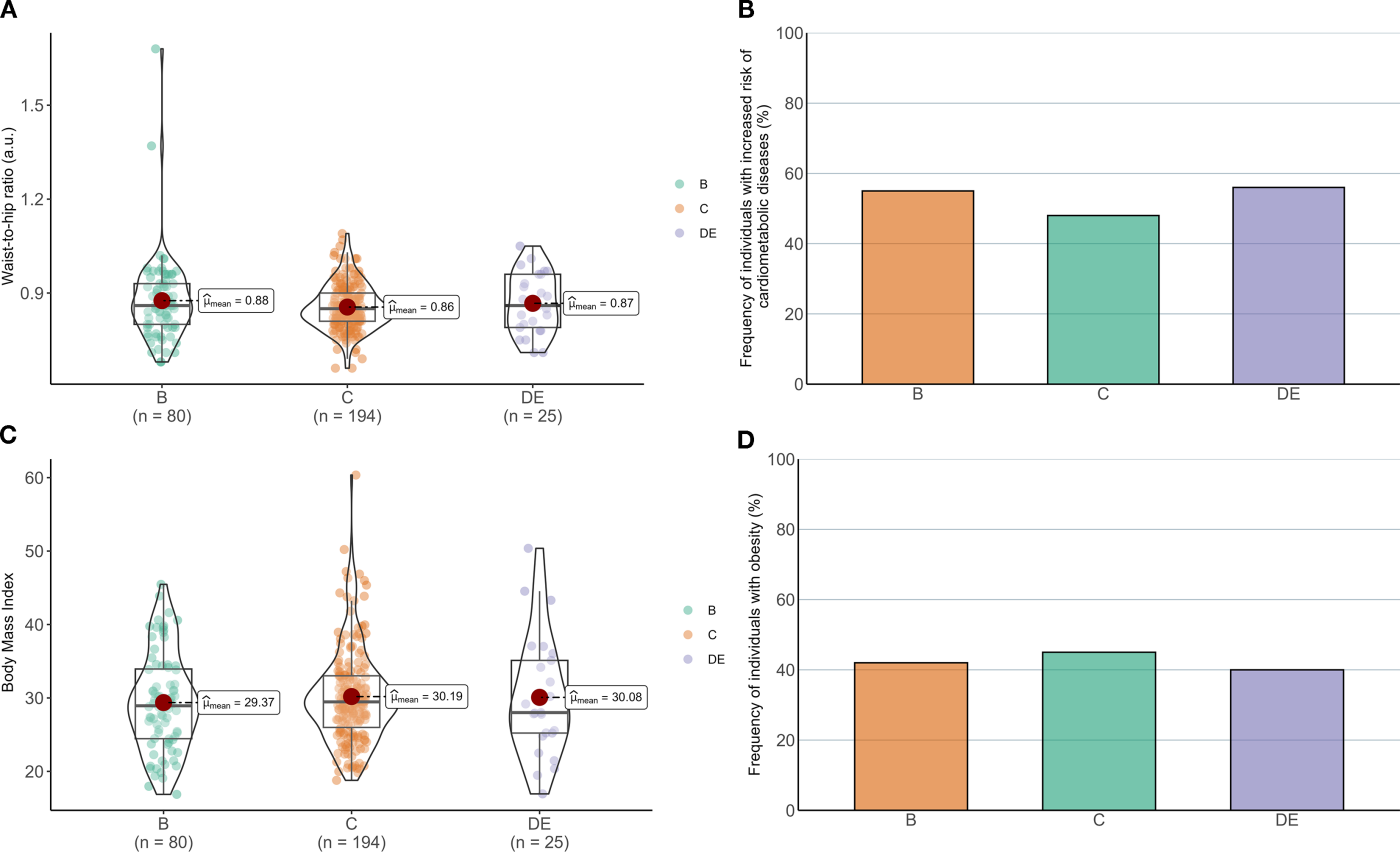
Body mass index and waist-to-hip ratio according to socioeconomic status. Panel **(A)** indicates body mass index in individuals according to socioeconomic status; Panel **(B)** indicates the frequency of individuals having obesity (body mass index ≥ 30) according to socioeconomic status; Panel **(C)** indicates waist-to-hip ratio in individuals according to socioeconomic status; Panel **(D)** indicates the frequency of individuals having increased risk of cardiometabolic diseases according to socioeconomic status.

The prevalence of minimal, mild, moderate, and severe anxiety and depressive symptoms, increased risk of cardiometabolic diseases and poor quality of life in each domain are presented in **Table 2**. The analysis revealed equivalent scores of depressive [B: 8.86 a.u.; C: 8.30 a.u.; DE: 5.68 a.u., P = 0.1570] and anxiety symptoms [B: 7.30 a.u.; C: 7.96 a.u.; DE: 7.60 a.u., P = 0.6440] across different socioeconomic status (**Figures 1A, C**). No statistically significant differences were observed among different socioeconomic status for frequency of moderate/severe depressive symptoms [B: 11%; C: 7%; DE: 4%, P = 0.3371] and anxiety symptoms [B: 8%; C: 6%; DE: 4%, P = 0. 6886] (**Figures 1B, D**). Similarly, we did not observe significant differences among different socioeconomic status for waist-to-hip ratio [B: 0.88 a.u.; C: 0.86 a.u.; DE: 0.87 a.u., P = 0.9920] and BMI [B: 29.37 kg/m^2^; C: 30.19; DE: 30.08, P = 0.7100] (**Figures 2A, C**). The frequency of individuals having increased risk of cardiometabolic diseases [B: 55%; C: 48%; DE: 56%, P = 0. 3507] and obesity [B: 42%; C: 45%; DE: 40%, P = 0. 8276] were similar among distinct socioeconomic status (**Figures 2B, D**).

**Table 2:** Prevalence of minimal, mild, moderate and severe symptoms of depression and anxiety, increased cardiometabolic risk and poor quality of life.

Outcomes	n = 299
Symptoms of depression	
Minimal	242 (81%)
Mild	34 (11%)
Moderate	17 (6%)
Severe	6 (2%)
Symptoms of Anxiety	
Minimal	173 (58%)
Mild	105 (35%)
Moderate	17 (6%)
Severe	4 (1%)
Increased cardiometabolic risk*	151 (50%)
Poor quality of life, score < 50	
Physical domain	18 (6%)
Psychological domain	11 (4%)
Social domain	26 (9%)
Environment domain	35 (12%)

**Figure 3:**
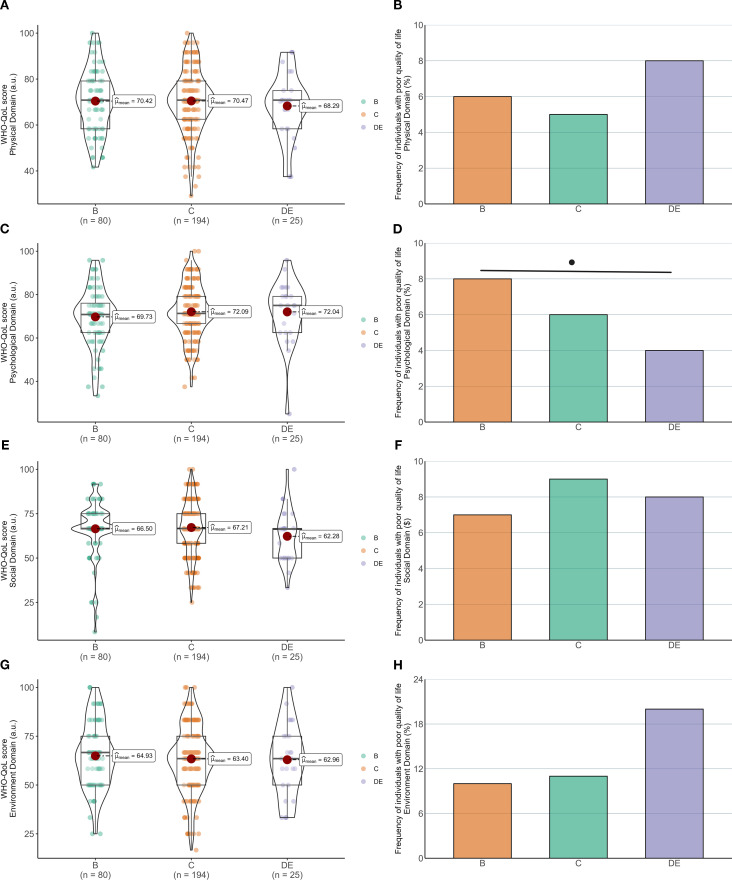
Quality of life in each domain according to socioeconomic status. Panel **(A)** indicates quality of life in the physical domain according to socioeconomic status; Panel **(B)** indicates the frequency of individuals having poor quality of life in the physical domain according to socioeconomic status; Panel **(C)** indicates quality of life in the psychological domain according to socioeconomic status; Panel **(D)** indicates the frequency of individuals having poor quality of life in the psychological domain according to socioeconomic status; Panel **(E)** indicates quality of life in the social domain according to socioeconomic status; Panel **(F)** indicates the frequency of individuals having poor quality of life in the social domain according to socioeconomic status; Panel **(G)** indicates quality of life in the environment domain according to socioeconomic status; Panel **(H)** indicates the frequency of individuals having poor quality of life in the environment domain according to socioeconomic status. *Indicates significant association. a.u., arbitrary units.

The quality of life in physical [B: 70.42 a.u.; C: 70.47 a.u.; DE: 68.29 a.u., P = 0.7390], psychological [B: 69.73 a.u.; C: 72.09 a.u.; DE: 72.04 a.u., P = 0.3580], social [B: 66.50 a.u.; C: 67.21 a.u.; DE: 62.28 a.u., P = 0.3150] and environment [B: 64.93 a.u.; C: 63.40 a.u.; DE: 62.96 a.u., P = 0.7540] domains were comparable among different socioeconomic status (**Figures 3A, C, E, G**). The analysis of quality of life in the psychological domain revealed a significant association, indicating that individuals living in moderate social vulnerability classified in higher socioeconomic status had a higher frequency of poor psychological quality of life compared with those in lower socioeconomic status (B: 8%; C: 6%; DE: 4%, P = 0.0157). In contrast, no statistically significant differences were verified among different socioeconomic status for individuals reporting poor quality of life in the physical (B: 6%; C: 5%; DE: 8%, P = 0.8946), social (B: 7%; C: 9%; DE: 8%, P = 0.8859) and environment domains (10%; C: 11%; DE: 20%, P = 0. 3839) (**Figures 3B, D, F, H**).

Linear regression analyses showed that, compared with participants in socioeconomic status B, those in status C and DE did not differ significantly in Beck Depression Inventory score, Beck Anxiety Inventory score, BMI, waist-to-hip ratio, or quality of life scores in the physical, psychological, social, and environmental domains (all P>0.05). These results remained unchanged after adjustment for age, ethnicity, sex, marital status, hypertension, and type 2 diabetes (all P>0.05). **Table 3** presents the regression analyses examining the associations between socioeconomic status and health outcomes. After false discovery rate correction for multiple comparisons, no statistically significant associations were detected between socioeconomic status and the assessed health outcomes.

**Table 3:** Linear regression analyses of the association between socioeconomic status and health outcomes.

Beck depression inventory score						
	Unadjusted model			Adjusted model ^a^		
Socioeconomic stratum (reference: B)	β	95%CI	P value	β	95%CI	P value
C	-0.56	-2.46 – 1.33	0.559	-0.26	-2.12 – 1.61	0.787
DE	-3.18	-6.45 – 0.08	0.056	-2.63	-5.86 – 0.60	0.110
Beck anxiety inventory score						
	Unadjusted model			Adjusted model ^a^		
Socioeconomic stratum (reference: B)	β	95%CI	P value	β	95%CI	P value
C	0.66	-0.74 – 2.06	0.355	-0.26	-2.12 – 1.61	0.787
DE	0.30	-2.11 – 2.71	0.807	-2.63	-5.86 – 0.60	0.110
Body mass index						
	Unadjusted model			Adjusted model ^a^		
Socioeconomic stratum (reference: B)	β	95%CI	P value	β	95%CI	P value
C	0.73	-1.02 – 2.48	0.414	0.68	-1.06 – 2.43	0.440
DE	0.71	-2.31 – 3.73	0.644	1.58	-1.45 – 4.60	0.306
Waist-to-hip ratio						
	Unadjusted model			Adjusted model ^a^		
Socioeconomic stratum (reference: B)	β	95%CI	P value	β	95%CI	P value
C	0.01	-0.04 – 0.04	0.904	0.01	-0.04 – 0.04	0.886
DE	0.01	-0.07 – 0.07	0.984	0.01	-0.06 – 0.08	0.816
Quality of life - physical domain						
	Unadjusted model			Adjusted model ^a^		
Socioeconomic stratum (reference: B)	β	95%CI	P value	β	95%CI	P value
C	0.06	-3.44 – 3.55	0.974	-0.58	-4.05 – 2.88	0.740
DE	-2.13	-8.16 – 3.90	0.487	-3.09	-9.09 – 2.91	0.311
Quality of life - psychological domain						
	Unadjusted model			Adjusted model ^a^		
Socioeconomic stratum (Reference: B)	β	95%CI	P value	β	95%CI	P value
C	2.36	-0.92 – 5.64	0.158	2.03	-1.29 – 5.35	0.229
DE	2.31	-3.35 – 7.96	0.422	1.90	-3.85 – 7.65	0.516
Quality of life - social domain						
	Unadjusted model			Adjusted model ^a^		
Socioeconomic stratum (reference: B)	β	95%CI	P value	β	95%CI	P value
C	0.71	-3.28 – 4.69	0.728	0.20	-3.78 – 4.19	0.920
DE	-4.22	-11.10 – 2.65	0.227	-6.05	-12.96 – 0.85	0.085
Quality of life – environmental domain						
	Unadjusted model			Adjusted model ^a^		
Socioeconomic stratum (reference: B)	β	95%CI	P value	β	95%CI	P value
C	-1.54	-5.82 – 2.75	0.480	-1.95	-6.21 – 2.30	0.367
DE	-1.98	-9.37 – 5.41	0.599	-3.79	-11.16 – 3.58	0.313

^a^Linear regression models were adjusted by age (as continuous), ethnicity [white, black and pardo], sex [male or female], marital status [single, married, divorced and widowed], hypertension [yes or no], and type II diabetes [yes or no].

## Discussion

This study examined whether socioeconomic status was associated with anxiety symptoms, depressive symptoms, BMI, waist-to-hip ratio, and quality of life among Brazilian adults living in areas of moderate social vulnerability. In the categorical analyses, quality of life in the psychological domain was significantly associated with socioeconomic status, with a higher frequency of poor psychological quality of life observed among individuals in higher socioeconomic strata. However, in the linear regression analyses, no statistically significant associations were detected between socioeconomic status and depressive symptoms, anxiety symptoms, BMI, waist-to-hip ratio, or quality of life scores in the physical, psychological, social, and environmental domains. These findings were consistent in both unadjusted models and models adjusted for age, ethnicity, sex, marital status, hypertension, and type 2 diabetes. Regarding the prevalence of health outcomes, the results indicate that: 8% and 7% of participants exhibited severe or moderate anxiety and depressive symptoms, respectively*;* 50% of the individuals presented an increased cardiometabolic risk; and the prevalence of individuals reporting poor quality of life in the physical, psychological, social, and environmental domains was 6%, 4%, 9%, and 12%, respectively.

Individuals living in conditions of social vulnerability are frequently exposed to economic, social, environmental, and psychosocial stressors, which may adversely affect anxiety and depressive symptoms. Consistent with this, previous research has reported elevated rates of mental disorders among socially vulnerable populations. For instance, the prevalence of depression among Brazilian adults living in socially vulnerable conditions such as urban slums or remote regions has been reported to range from approximately 8% to over 19% (13, 32, 33). Similarly, the prevalence of common mental disorders, including anxiety and depression, reaches 14.7% in socioeconomically disadvantaged urban populations (13). In our study, 8% and 7% of Brazilian adults living in moderate social vulnerability exhibited severe or moderate anxiety and depressive symptoms, respectively. Together, these findings underscore the disproportionate burden of mental health issues among individuals experiencing social and economic disadvantages.

Excess mortality among individuals with mental disorders extends well beyond the commonly recognized causes, such as suicide and accidental injuries, encompassing both communicable and non-communicable diseases (7, 8, 11). Notably, cardiometabolic diseases have been identified as a major contributor to premature death in this population (7, 10). Our findings revealed a high prevalence of obesity (44%), hypertension (44%), and an increased risk of cardiometabolic diseases (50%) among individuals living in moderate social vulnerability. These findings are consistent with previous studies reporting a high prevalence of obesity and increased waist circumference among socially vulnerable individuals (16, 17). These results reinforce the need for targeted public health interventions aimed at early detection and management of cardiometabolic risk factors within socially vulnerable populations to reduce preventable morbidity and premature mortality (18). However, cardiometabolic risk was assessed only through anthropometric indicators and self-reported hypertension and diabetes, without laboratory biomarkers, objective blood pressure measurements, lipid profile, glycemic markers, or inflammatory markers. Therefore, these findings should not be interpreted as a comprehensive clinical assessment of cardiometabolic risk.

Given the high prevalence of anxiety and depressive symptoms, obesity and increased risk of cardiometabolic diseases assessed by waist-to-hip ratio among individuals living in social vulnerability, it is reasonable to assume that these individuals also experience poor quality of life. Indeed, our data support this assumption, as we observed that the prevalence of individuals reporting poor quality of life in the physical, psychological, social, and environmental domains was 6%, 4%, 9%, and 12%, respectively. These findings highlight the need for comprehensive health and social policies that tackle the broader social determinants (e.g., social and environmental factors) influencing quality of life among socially vulnerable populations.

Although socially vulnerable populations are already exposed to various stressors, we sought to examine whether socioeconomic status is significantly associated with health outcomes. For this purpose, participants were classified using a standardized instrument widely employed in Brazil to assess household economic status. Briefly, this tool estimates the socioeconomic level of families using information about ownership of durable goods (e.g., television, refrigerator, car), access to services (e.g., presence of a domestic worker) and, the education level of the head of the household, thereby allowing individuals to be categorized into economic status A, B, C and DE. Our initial analysis revealed the absence of individuals from socioeconomic status A, suggesting that these areas are largely avoided by more socioeconomically advantaged groups. However, socioeconomic status based on household assets and education may not fully capture the broader structural determinants that shape health among individuals living in social vulnerability. Factors such as employment insecurity, informal or unstable work, neighborhood violence, limited access to health care services, transportation barriers, food insecurity, and reduced social support may contribute independently to anxiety symptoms, depressive symptoms, adiposity-related indicators, and quality of life (15, 34–36). These factors may partially explain the limited differences observed across socioeconomic strata in the present sample, as individuals classified in strata B, C, and DE may still be exposed to similar adverse social and environmental conditions. Therefore, future studies should examine socioeconomic status together with these structural and contextual determinants to better understand how social vulnerability influences physical and mental health outcomes.

Regarding the relationship between socioeconomic status and health outcomes, quality of life in the psychological domain was significantly associated with socioeconomic status. Specifically, individuals in higher socioeconomic status had a higher frequency of poor psychological quality of life compared with those in lower socioeconomic status. However, in the linear regression analyses, no statistically significant associations were detected between socioeconomic status and depressive symptoms, anxiety symptoms, BMI, waist-to-hip ratio, or quality of life scores in the physical, psychological, social, and environmental domains. These findings were consistent in both unadjusted and adjusted models. The discrepancy between the categorical analysis of poor psychological quality of life and the continuous regression analysis of psychological quality-of-life scores suggests that this finding should be interpreted with caution. Moreover, because multiple statistical comparisons were performed, this association may represent a Type I error and should therefore be considered exploratory. This unexpected association may reflect the complex relationship between socioeconomic status and psychological well-being among individuals living in moderate social vulnerability. In this context, even individuals in higher socioeconomic status may remain exposed to shared environmental stressors and other unmeasured psychosocial factors, such as occupational stress, financial expectations, caregiving burden, or limited social support. Thus, the higher frequency of poor psychological quality of life in higher socioeconomic status should be interpreted cautiously and explored in future studies, including analyses of additional social and contextual factors individually, such as employment status, job insecurity, and the presence or absence of supportive family relationships.

This study has limitations. First, no participants were classified in socioeconomic status A, the highest category according to the Brazilian Economic Classification Criteria. Therefore, the findings do not allow comparisons across the full socioeconomic spectrum and should not be generalized to individuals from the highest socioeconomic status. Rather, the results should be interpreted as describing intra-group socioeconomic variation among participants classified in strata B, C, and DE living in areas of moderate social vulnerability. Second, although the total sample size was adequate for the planned analyses, some subgroup comparisons included small numbers of participants, particularly in the DE status, which may have reduced statistical power to detect modest differences across socioeconomic groups. Thus, nonsignificant findings should be interpreted as the absence of statistically significant associations detected in this sample, rather than evidence of no association. Third, the cross-sectional design precludes causal inference. Fourth, the small, non-probabilistic sample limits generalizability to the broader Brazilian population. In addition, the predominance of female participants may introduce sex-specific bias and limit generalizability to men. Recruitment through university community programs and social media may also have introduced selection bias, as participants reached through these channels may differ from the broader population living in moderate social vulnerability. Finally, depressive and anxiety symptoms were assessed using self-report questionnaires rather than clinical diagnostic interviews, and questionnaire-based measures are subject to recall bias and underreporting.

In conclusion, among Brazilian adults living in areas of moderate social vulnerability and classified within socioeconomic status B, C, and DE, socioeconomic status was not significantly associated with anxiety and depressive symptoms, BMI, waist-to-hip ratio, or quality of life. These findings suggest that, within this moderately vulnerable population, intra-group socioeconomic differences were not associated with several health indicators in the present sample. Because no participants were classified in stratum A, these results should not be generalized to individuals from the highest socioeconomic status or interpreted as evidence that high socioeconomic status does not protect against poor health in the broader population. Future studies should investigate whether shared environmental and social stressors may attenuate socioeconomic gradients in health outcomes among populations living in conditions of social vulnerability.

## Data Availability

The raw data supporting the conclusions of this article will be made available by the authors, without undue reservation.
